# Immunomodulatory and clinical effects of receptor-interacting protein kinase 1 (RIPK1) inhibitor eclitasertib (SAR443122) in patients with severe COVID-19: a phase 1b, randomized, double-blinded, placebo-controlled study

**DOI:** 10.1186/s12931-024-02670-z

**Published:** 2024-02-28

**Authors:** Pierre-Francois Clot, Christine Farenc, Benjamin T. Suratt, Tillmann Krahnke, Agnes Tardat, Peter Florian, Robert Pomponio, Naimish Patel, Maria Wiekowski, Yong Lin, Benjamin Terrier, Heribert Staudinger

**Affiliations:** 1 Translational Medicine and Early Development (TMED)/Clinical Pharmacology (TMCP) and Neuro and Neuro-Immunology, 371 Rue du Professeur Blayac, Sanofi, Montpellier, 34080 France; 2 TMED Pharmacokinetics Dynamics and Metabolism, Sanofi, Montpellier, France; 3 Early Clinical Development Immunology and Inflammation, Sanofi, Cambridge, MA United States of America; 4 CEP Germany, Sanofi Deutschland GmbH, Frankfurt, Germany; 5 Early Development Operations, Sanofi, Montpellier, France; 6 Type 1/17 Immunology and Arthritis, Sanofi Deutschland GmbH, Frankfurt, Germany; 7 TMED Biomarkers and Clinical Bioanalysis, Sanofi, Framingham, MA United States of America; 8 Global Development in Immunology and Inflammation, Sanofi, Cambridge, MA United States of America; 9 Immunology and Inflammation Development Franchise, Sanofi, Bridgewater, NJ United States of America; 10 Sanofi, Bridgewater, NJ United States of America; 11 Department of Internal Medicine, Cochin Hospital, Paris, France; 12 Present Address: Head of Fibrotic Disease Research, Boehringer Ingelheim Vetmedica GmbH, Global AH Research, Ingelheim, Germany

**Keywords:** Biomarker, Inflammation, Immunomodulatory, Receptor-interacting serine/threonine protein kinase 1, COVID-19

## Abstract

**Background:**

Targeting receptor-interacting serine/threonine protein kinase 1 could mitigate the devastating sequelae of the hyperinflammatory state observed in severe cases of COVID-19. This study explored the immunomodulatory and clinical effects of the receptor-interacting serine/threonine protein kinase 1 inhibitor SAR443122 (eclitasertib) in patients with severe COVID-19.

**Methods:**

In this Phase 1b, double-blinded, placebo-controlled study (NCT04469621) a total of 82 patients were screened, of whom 68 patients were eligible and randomized (2:1) to receive eclitasertib 600 mg (300 mg twice daily) or placebo up to 14 days. Primary outcome was relative change in C-reactive protein from baseline to Day 7. Time to clinical improvement using 7-point ordinal scale, ventilator/respiratory failure-free days, change in SpO_2_/FiO_2_ ratio, and biomarkers of severe COVID-19 were explored.

**Results:**

Geometric mean ratio (point estimate [90% confidence interval]) of the relative change from baseline in C-reactive protein with eclitasertib vs. placebo on Day 7 was 0.85 (0.49–1.45; *p* = 0.30). Median time to 50% decrease in C-reactive protein from baseline was 3 days vs. 5 days (*p* = 0.056) with eclitasertib vs. placebo. Median time to ≥ 2-point improvement on 7-point clinical symptoms scale was 8 days vs. 10 days with eclitasertib vs. placebo (*p* = 0.38). Mean ventilator/respiratory failure-free days, change in baseline-adjusted SpO_2_/FiO_2_ ratio, and clinical biomarkers showed consistent numerical improvements with eclitasertib vs. placebo. The most frequently reported treatment-emergent adverse events were gastrointestinal disorders and condition aggravated/worsened COVID-19 pneumonia.

**Conclusions:**

Eclitasertib was well tolerated with consistent trends toward more rapid resolution of inflammatory biomarkers and clinical improvement in severe COVID-19 patients than placebo.

**ClinicalTrials.gov identifier:**

NCT04469621, first posted on clinicaltrials.gov on July 14, 2020.

**Supplementary Information:**

The online version contains supplementary material available at 10.1186/s12931-024-02670-z.

## Background

Coronavirus disease 2019 (COVID-19) is caused by severe acute respiratory syndrome coronavirus 2 (SARS-CoV-2), with influenza-like initial symptoms such as cough, fever, fatigue, headache, myalgias, and diarrhoea [1]. Patients with COVID-19 may become critically ill with acute respiratory distress syndrome (ARDS), which typically begins approximately 7–10 days after symptom onset [2] and is reported to occur in 29% of severe COVID-19 cases during the second week of hospitalisation [3]. Hyperinflammation in COVID-19, characterised by reactive hemophagocytic lymphohistiocytosis, may cause cytopenia, coagulopathy, tissue damage, liver injury, and macrophage activation [4–8]. The overwhelming production of inflammatory cytokines causes organ dysfunction and, eventually, death [9]. Several therapeutic interventions have been proposed to mitigate this inflammatory organ injury in viral pneumonia, and the value of glucocorticoids has been widely debated [10].

Receptor-interacting serine/threonine-protein kinase 1 (RIPK1) is an intracellular protein that regulates the downstream signalling of tumour necrosis factor receptor 1 (TNFR1), toll-like receptors (TLRs) 3 and 4, and interferon receptors (IFNRs), by exhibiting both kinase activity-dependent and kinase activity-independent functions [11]. RIPK1-mediated signalling promotes inflammation and induces apoptotic or necroptotic cell death [12–15]. Both RIPK1 kinase-driven inflammation and cell death are key contributors to tumour necrosis factor-alpha (TNF-α)-induced systemic inflammatory response syndrome (SIRS) [16–19]. Moreover, RIPK1 kinase inhibition may suppress vascular dysfunction, endothelial/epithelial cell damage and exacerbated inflammatory signalling [16, 20]. It could complement antiviral therapy by inhibiting the inflammatory surge and necroptosis of pulmonary epithelial cells [21, 22], preventing, or reducing the effect of severe inflammation on respiratory function and other organ failure. Since RIPK1 is considered a master regulator of proinflammatory cell death, selectively targeting its kinase activity was hypothesised to mitigate the devastating sequelae of the hyperinflammatory state observed in late-stage severe COVID-19.

SAR443122 (eclitasertib) is a highly potent, selective oral inhibitor of RIPK1 kinase activity under development as an immunomodulatory drug for cutaneous lupus erythematosus and ulcerative colitis. We hypothesised that the use of eclitasertib to target patients with severe COVID-19 at an increased risk of SIRS would reduce inflammatory signalling and improve clinical outcomes. This double-blind, placebo-controlled, phase 1b study evaluated the immunomodulatory and clinical effect of eclitasertib vs. placebo in adult patients hospitalised with severe COVID-19.

## Methods

### Study design and patients

This multinational, multi-center, double-blind, placebo-controlled, randomized study enrolled adult patients hospitalised with severe COVID-19 (NCT04469621, first posted on clinicaltrials.gov on July 14, 2020). The study included three periods: screening period (up to 4 days), treatment period (up to 14 days + one end-of-treatment [EoT] day), and post-intervention observation period (up to 13 days) (Supplementary Fig. 1). Hospitalised adults (18–80 years) with severe COVID-19 infection, confirmed by RT-PCR, or other commercial or public health assay, who had laboratory signs consistent with systemic inflammation (C-reactive protein [CRP] > 50 mg/L) and required oxygen supplementation were enrolled in this study. Patients were excluded from enrolment if they (a) were unlikely to survive 48 h according to the investigator; (b) required the use of invasive or non-invasive positive pressure ventilation or more than 40% fraction of inhaled oxygen (FiO_2_) and more than 6 L/min of oxygen flow rate; (c) had significant liver enzyme abnormalities, thrombocytopenia, or anaemia; (d) were receiving immunomodulatory therapies (including, but not limited to, anti-IL-6, anti-IL-6R antagonists, Janus kinase inhibitors inhibitors, B-cell depleting agents, anakinra, abatacept, TNF inhibitors, alkylating agents cyclosporine, azathioprine, mycophenolate mofetil, methotrexate, intravenous immunoglobulin or convalescent serum), and/or chronic systemic corticosteroids at a dose higher than prednisone 10 mg or equivalent, for a non-COVID-19-related condition; (e) were pregnant or breastfeeding; or (f) had tuberculosis/non-tuberculous mycobacterial infections or suspected/known active systemic bacterial or fungal infections.

The study was approved by the Institutional Review Board/Institutional Ethics Committee of each study site (Supplementary Table 1), and the study was performed according to consensus ethics principles derived from international ethics guidelines, including the Declaration of Helsinki and the International Council for Harmonisation of Technical Requirements for Pharmaceuticals for Human Use (ICH) guidelines for Good Clinical Practice (GCP), all applicable laws, rules, and regulations. A signed written informed consent form was obtained from each patient before conducting any study-related procedures.

### Procedure

Enrolled patients were randomized (stratified by site) using an interactive response technology (IRT) system in a 2:1 ratio to receive either eclitasertib 600 mg or a matching placebo daily for 14 days or up to discharge from the hospital, whichever came first. Each hospitalised patient was evaluated daily based on various factors including supplemental oxygen use, and the clinical status of each patient was assessed using a 7-point ordinal scale as follows: 1 = death; 2 = hospitalised, on invasive mechanical ventilation or extracorporeal membrane oxygenation (ECMO); 3 = hospitalised, on non-invasive ventilation or high-flow oxygen devices; 4 = hospitalised, requiring supplemental oxygen; 5 = hospitalised, not requiring supplemental oxygen – requiring ongoing medical care (COVID-19 related or otherwise); 6 = hospitalised, not requiring supplemental oxygen – no longer requires ongoing medical care; and 7 = not hospitalised. Concomitant therapy including thrombolytic therapy and vasopressor treatment was recorded.

Biomarker assessments included clinical laboratory variables (CRP, laboratory markers of severe COVID-19 [D-dimer] [23], and haematology parameters [white blood cell count, differential blood lymphocytes, and neutrophil/lymphocyte ratio]). Blood samples for laboratory assessments, including haematology and quantification of CRP and D-dimer levels, were collected on Days 1 (pre-dose), 3, 5, 7, and 15. Blood samples for lactate dehydrogenase (LDH) and ferritin were collected on Day 1 (pre-dose) and then as and when available per clinical care. For pharmacodynamic assessment, blood samples for cytokine and chemokine biomarker analysis were collected on Days 1 (pre-dose), 3, 5, 7, and 15. The quantitative viral load of SARS-CoV-2 was measured at baseline and on Days 3, 7, and 15/ EoT using reverse transcription-polymerase chain reaction.

The assessment of pharmacokinetics (PK) included the measurement of eclitasertib plasma concentrations (using a validated liquid chromatography–mass spectrometry [LC/MS] method) over 2 weeks of treatment at selected timepoints: within 2–5 h after the first morning dose (around maximum concentration [C_max_]) on Day 1, before the morning dosing on Day 3 (trough concentration [C_trough_]), and before the morning dose and within 2–5 h after the morning dose on Days 7 and 14. Moreover, a PK sample was obtained within 1 h before discharge when this occurred before Day 14. Individual PK parameters such as C_max_, C_trough_, and area under the curve from 0 to 12 h (AUC_0–12 h_) were calculated using a Maximum a posteriori Bayesian estimation and a population PK model.

### Outcomes

The primary endpoint was a relative change in CRP level from baseline to Day 7, defined as the ratio of CRP level on Day 7 vs. CRP level at baseline. The key secondary endpoints included: (a) time to 50% decrease in CRP level from baseline; (b) time to improvement in oxygenation as measured by oxygen saturation ≥ 92% breathing in room air over 48 h or until discharge; and (c) change in peripheral blood oxygen saturation/fraction of inspired oxygen (SpO_2_/FiO_2_) ratio from baseline to Day 7. Other secondary endpoints included: (a) number of days without the need for oxygen support and alive within 28 days post-randomization (defined as any calendar day with oxygen saturation ≥ 92% breathing in room air) up to Day 28; (b) number of ventilator-free and respiratory failure-free days (VFDs/RFFDs) and alive up to Day 28; (c) incidence of death up to Day 28; (d) percentage of patients receiving thrombolytic and vasopressor treatment up to Day 28; and (e) change in inflammatory biomarkers (white blood cell count, blood neutrophil and lymphocytes counts, and neutrophil/lymphocyte ratio), and markers of severe COVID-19 (D-dimer) from baseline to Day 7 and at EoT. The exploratory endpoints included: (a) change in ferritin and LDH from baseline to Day 7 and at EoT; (b) time to clinical improvement on the 7-point ordinal scale; (c) change in peripheral cytokine and biomarker levels from baseline up to EoT; (d) quantitative SARS-CoV-2 viral load in blood at baseline and on Days 3, 5, and 7 and at EoT; and (e) eclitasertib plasma pharmacokinetic (PK) parameters (for C_max_, area under the curve over the dosing interval [AUC_0–12 h_], and C_trough_). Safety was evaluated up to 28 days post the initial dosing. Adverse event coding was performed using the Medical Dictionary for Regulatory Activities version 23.1.

VFDs were defined as the number of calendar days within the 28 days after randomization for which the patient was alive and without the use of invasive mechanical ventilation or non-invasive mechanical ventilation or extracorporeal life support. RFFDs were defined as the number of days for which the patient was alive and not on invasive mechanical ventilation, non-invasive mechanical ventilation, or high-flow nasal cannula at ≥ 50% FiO_2_ and ≥ 30 L/min of oxygen flow during the 28-day study period.

### Statistical analyses

A sample size of 60 evaluable patients allocated in a 2:1 ratio was required for a t-test to achieve an overall power of about 80% at one-sided significance level alpha = 0.05 by assuming a standard deviation of 1.04 and a true difference between treatment groups in mean log-relative change from baseline in CRP of log (0.5) or equivalently a 50% reduction in geometric mean relative to placebo. The efficacy population included all randomized patients who received at least one complete dose of the study drug with at least one post-study drug administration measurement and without a major protocol deviation. Safety population included all randomized patients exposed to the study drug. The PK analysis population included all patients who received any study drug and who had at least one non-missing result following the first dose of the study drug. For the primary analysis, the relative change in CRP from baseline was analysed using a linear mixed model with repeated measurements (MMRM) fitted on log-relative change from baseline for Days 3, 5, 7, and 15.

Kaplan-Meier analysis was performed to determine the following variables: (a) time to 50% decrease in CRP level from baseline; (b) time to improvement in oxygenation as measured by oxygen saturation ≥ 92% breathing in room air over 48 h or until discharge; and (c) time to improvement by at least two points on the 7-point clinical scale. Treatment arms were compared in an exploratory fashion using the log-rank test.

For the change from baseline in SpO_2_/FiO_2_ ratio, a linear MMRM was fitted based on observed values for Days 2 through 7 and 15. For other secondary endpoints, data were summarised by treatment arms with descriptive statistics except for laboratory markers of inflammation and severe COVID-19.

The data were analysed using SAS® (Unix, Version 9.4, SAS Institute, NC USA) and R software (R Core Team [2018]). This study is registered with the ClinicalTrials.gov registry, NCT04469621.

## Results

**Fig. 1:**
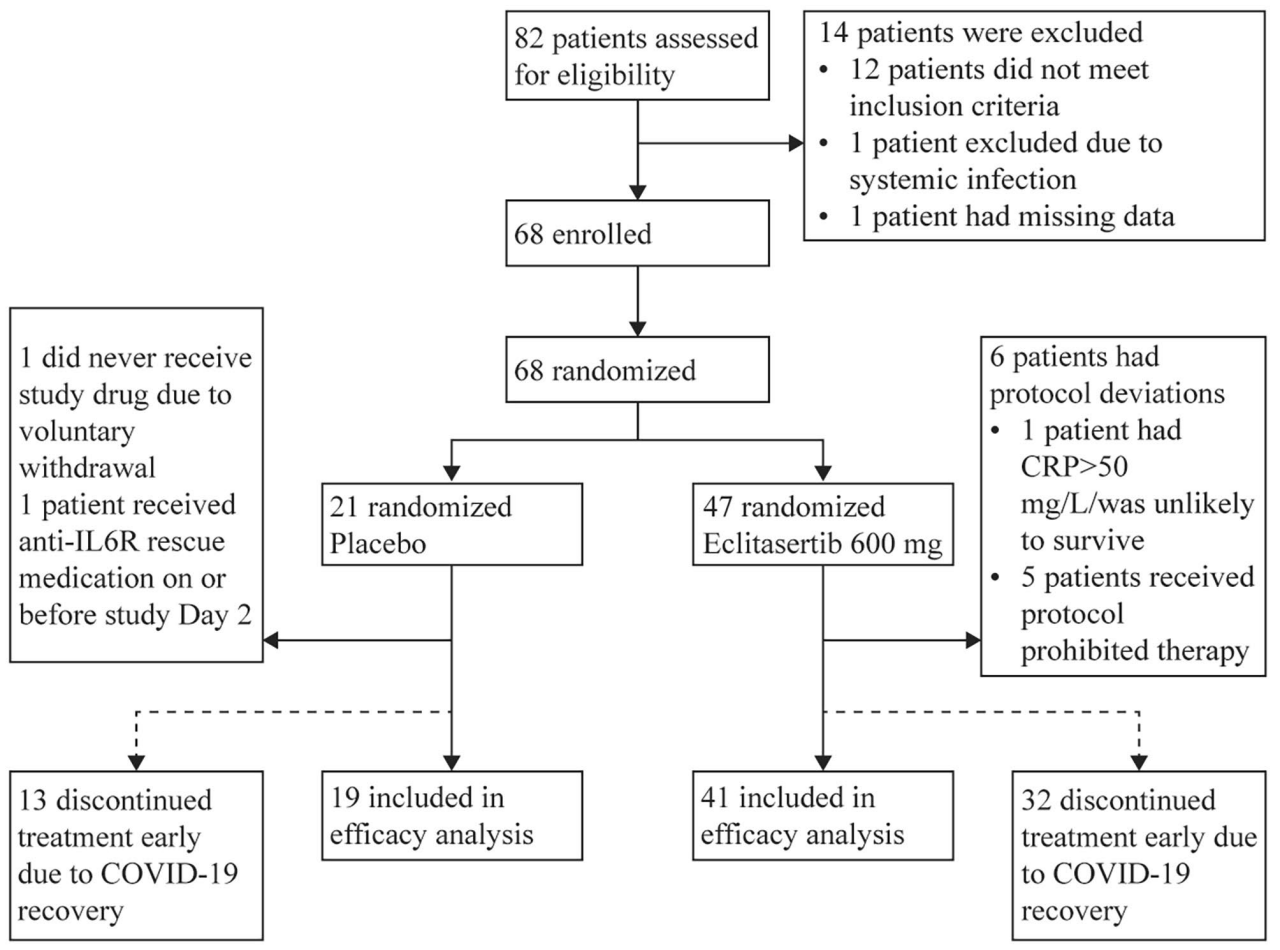
Trial profile

Between 17 July 2020 and 23 October 2020, 82 patients were screened in 10 centers in five countries (Argentina, Brazil, Chile, Mexico, and the Russian Federation), of whom 68 patients were eligible and randomized (Fig. 1). Data for 67 and 60 patients were available for the safety and efficacy analyses, respectively. On Day 7, data for only 57.9% (11 of 19 patients) and 48.8% (20 of 41 patients) were available in the placebo and treatment groups, respectively, mainly because the patients were discharged from the hospital after COVID-19 recovery. Demographic and other baseline characteristics including medical history profiles specific to this study were generally similar between the two treatment groups (Table 1), although the baseline mean CRP level was slightly higher in the placebo group than the eclitasertib group (133.5 mg/L vs. 105.6 mg/L). Corticosteroids, as the standard of care, were administered in 65% and 63.8% of the patients in the placebo and eclitasertib groups, respectively.

**Table 1:** Demographics and patient characteristics at baseline (safety population)

	Placebo (*N* = 20)	Eclitasertib 600 mg (*N* = 47)	All (*N* = 67)
**Age (years)**, mean (SD)	55.2 (13.5)	58.9 (11.3)	57.8 (12.0)
**Sex, n(%)**			
Male / Female	12 (60.0)/ 8 (40.0)	28 (59.6)/ 19 (40.4)	40 (59.7)/ 27 (40.3)
**Race, n(%)**			
White	16 (80.0)	40 (85.1)	56 (83.6)
Black or African American	2 (10.0)	3 (6.4)	5 (7.5)
American Indian or Alaska Native	1 (5.0)	1 (2.1)	2 (3.0)
Unknown	1 (5.0)	2 (4.3)	3 (4.5)
Multiple	0	1 (2.1)	1 (1.5)
American Indian or Alaska Native/White	0	1 (2.1)	1 (1.5)
**Ethnicity, n (%)**			
Hispanic or Latino	13 (65.0)	30 (63.8)	43 (64.2)
Not Hispanic or Latino	6 (30.0)	14 (29.8)	20 (29.9)
Not Reported	1 (5.0)	1 (2.1)	2 (3.0)
Unknown	0	2 (4.3)	2 (3.0)
**Baseline weight (kg)**, mean (SD)	88.9 (19.3)	89.1 (19.7)	89.1 (19.4)
**BMI (kg/m** ^**2**^ **), n(%)**			
18.5 to < 25	2 (10.0)	6 (12.8)	8 (11.9)
25 to < 30	8 (40.0)	20 (42.6)	28 (41.8)
30 to < 40	9 (45.0)	13 (27.7)	22 (32.8)
≥ 40	1 (5.0)	8 (17.0)	9 (13.4)
**Medical history group, n (%)**			
Obesity	10 (50.0)	22 (46.8)	32 (47.8)
Diabetes	4 (20.0)	17 (36.2)	21 (31.3)
Respiratory disorders	4 (20.0)	8 (17.0)	12 (17.9)
Renal disorders	1 (5.0)	7 (14.9)	8 (11.9)
Cardiovascular disorders	2 (10.0)	4 (8.5)	6 (9.0)
Autoimmune disorders	2 (10.0)	1 (2.1)	3 (4.5)
**Days since COVID-19 diagnosis**, mean (SD)	7.7 (3.8)	8.0 (4.9)	7.9 (4.5)
**Days since COVID-19 hospitalisation**, mean (SD)	3.1 (2.8)	2.8 (1.5)	2.9 (2.0)
**ICU admission at baseline**			
No / Yes	17 (85.0)/ 3 (15.0)	46 (97.9)/ 1 (2.1)	63 (94.0)/ 4 (6.0)
**Baseline CRP (mg/L)**, mean (SD)	133.5 (88.4)	105.6 (67.2)	113.9 (74.6)
**Baseline Ferritin (ug/L)**, mean (SD)*	1411.1 (1086.9)	2389.0 (8126.6)	2089.3 (6784.3)
**Baseline LDH (IU/L)**, mean (SD)*	338.4 (116.0)	350.8 (111.4)	347.0 (112.0)
**Baseline SpO**_**2**_**/FiO**_**2**_**(ratio)**, mean (SD)	294.1 (55.3)	296.8 (62.9)	296.0 (60.3)
**Oxygen therapy at baseline**			
Nasal cannula	13 (65.0)	25 (53.2)	38 (56.7)
Simple face mask	6 (30.0)	17 (36.2)	23 (34.3)
Non-rebreather face mask	0	1 (2.1)	1 (1.5)
High-flow nasal cannula	1 (5.0)	1 (2.1)	2 (3.0)
Non-invasive ventilation	0	1 (2.1)	1 (1.5)
Ambient	0	2 (4.3)	2 (3.0)
**Steroid treatment**			
Dexamethasone	13 (65.0)	29 (61.7)	42 (62.7)
Prednisone	0	4 (8.5)	4 (6.0)
Methylprednisolone	1 (5.0)	1 (2.1)	2 (3.0)

BMI: Body mass index; CRP: C-reactive protein; ICU: Intensive care unit; LDH: Lactate Dehydrogenase; N: Number of patients; SpO2/FiO2: Peripheral oxygen saturation/Fraction of inspired oxygen. * *N* = 19 for placebo and *N* = 43 for eclitasertib 600 mg

Note: Baseline is defined as the last available and evaluable value before the first administration of the Investigational Medicinal Product

**Fig. 2:**
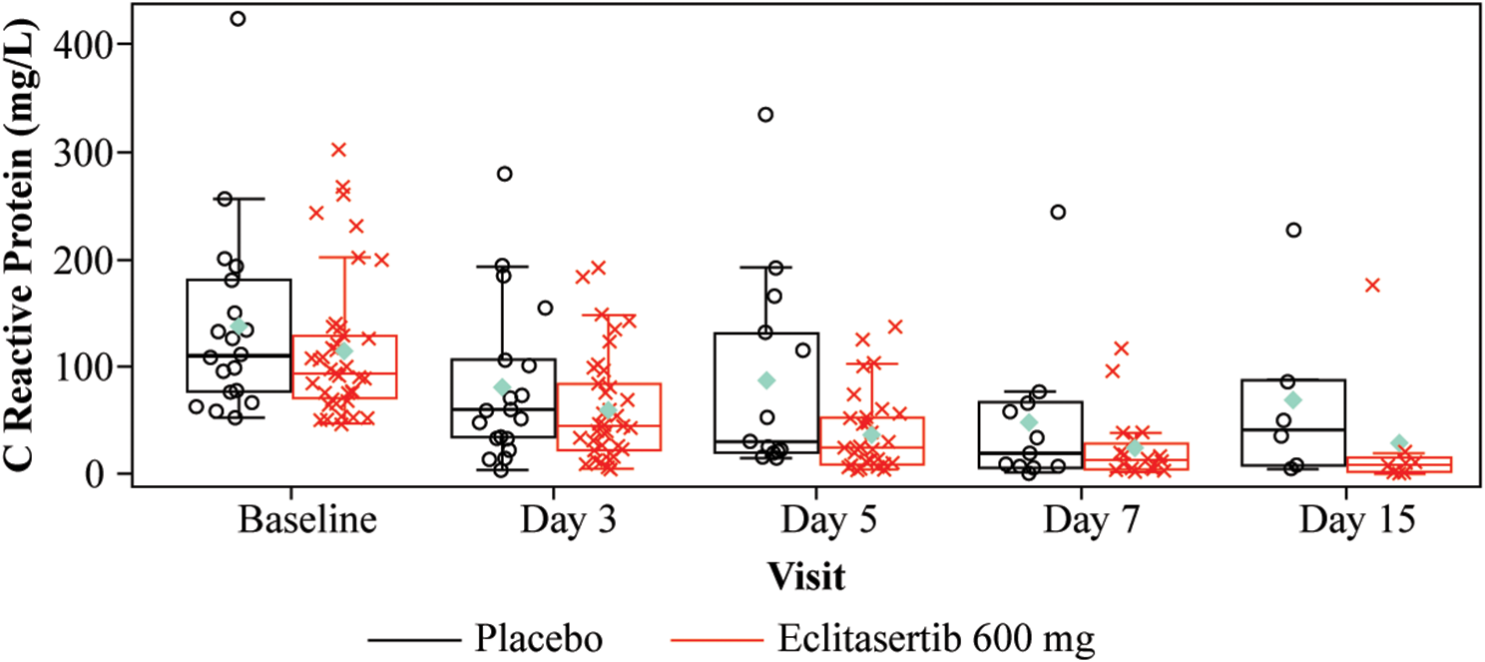
Boxplot of raw value of CRP over time (efficacy population).The teal diamond corresponds to the group arithmetic mean. The horizontal line in the box interior represents the group median. The length of the box represents the interquartile range (the distance between the 25^th^ and 75^th^ percentiles). The other symbols correspond to patient values

The primary analysis showed that the decrease in CRP from baseline was numerically greater in the eclitasertib than the placebo group on Days 3, 5, and 7 (Fig. 2). A similar trend was observed after imputing missing CRP values using last observation carried forward (LOCF) analysis. In the primary MMRM analysis (efficacy population), the ratio of the baseline-adjusted relative change in CRP in the eclitasertib vs. placebo group from baseline to Day 7 was 0.85 (90% CI: 0.49–1.45). Although not significant (*p* = 0.302), the decrease in CRP in the eclitasertib vs. placebo group from baseline to Day 7 tended to be rapid.

**Fig. 3﻿:**
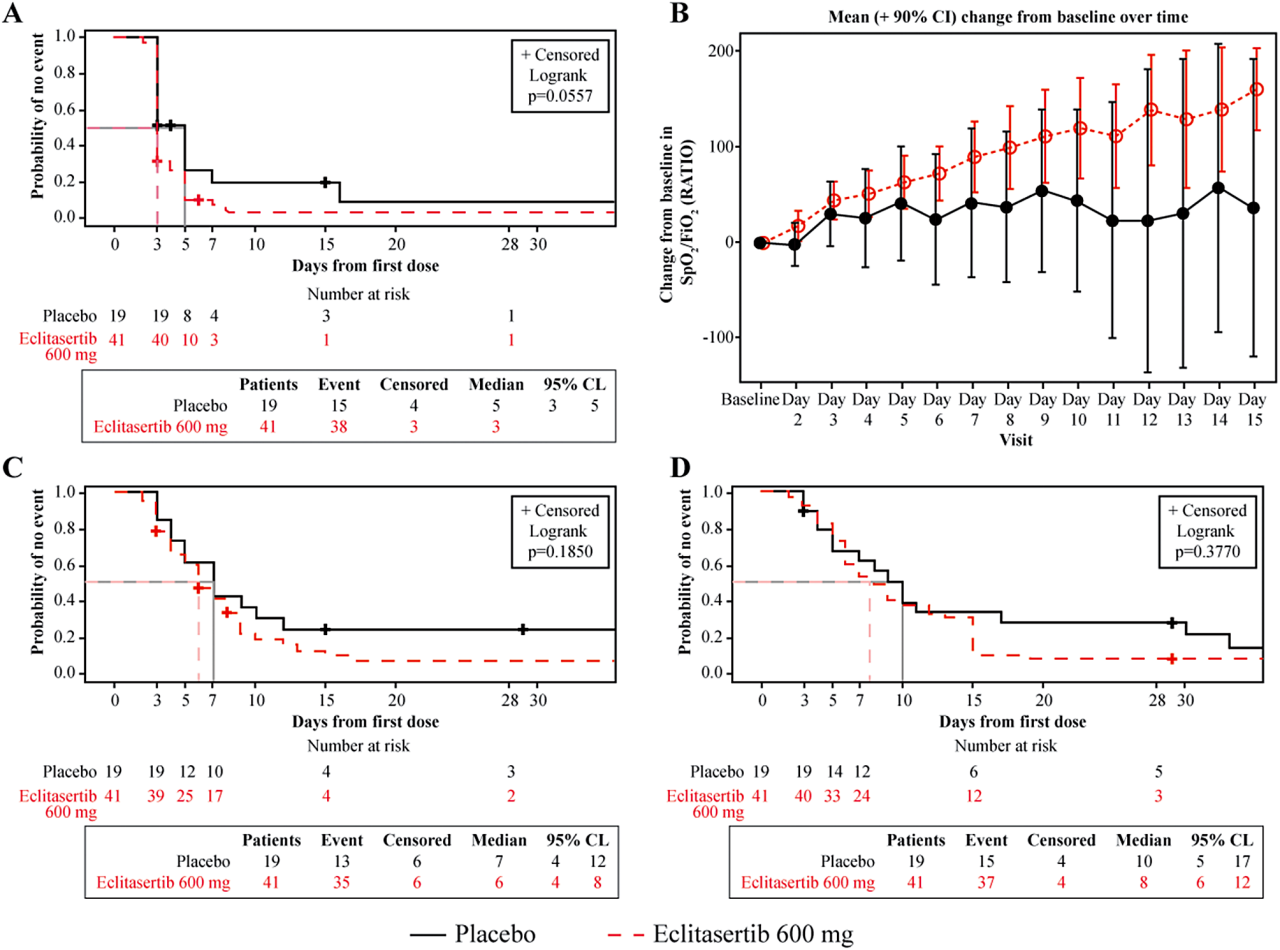
Time to 50% improvement in CRP levels (3 A), time to improvement of oxygenation (SpO_2_ /FiO_2_ ratio) (3B), time to liberation from supplemental oxygen (3 C), and improvement by at least two points on 7-point clinical scale (3D) for efficacy population. Event times for patients not meeting this criterion was censored at the last observation time point. For patients who died during the study without experiencing the event, the last observation collected is carried forward to the longest duration of follow-up for any patient plus 1 day. Event is defined as 50% decrease relative to baseline CRP level (3 A), presence of SpO_2_ ≥92% without use of any supplemental oxygen device on two consecutive days or on the day of discharge (3 C), and an improvement of at least 2 points in category of 7-point clinical scale from baseline (3D)

Kaplan-Meier curves for time to 50% improvement in CRP levels, improvement in oxygenation, time to liberation from supplemental oxygen, and improvement by at least two points in the category of 7-point scale are presented in Fig. 3. Median time to 50% decrease in CRP level from baseline was 3 vs. 5 days in the eclitasertib vs. placebo group (*p* = 0.056; Fig. 3A). Eclitasertib achieved faster improvement in oxygenation (SpO_2_/FiO_2_ ratio) than placebo (Fig. 3B). Time to liberation from supplemental oxygen was 6 vs. 7 days (*p* = 0.185) in the eclitasertib vs. placebo group, respectively (Fig. 3C). Median time for ≥ 2-point improvement on the 7-point scale was 8 vs. 10 days (*p* = 0.38) in the eclitasertib vs. placebo group (Fig. 3D).

Baseline-adjusted mean change in the SpO_2_/FiO_2_ ratio from baseline to Day 7 was numerically greater in the eclitasertib than the placebo group (Table 2). A similar result favouring eclitasertib over placebo was observed at all visits modelled using MMRM, with the largest increase observed on Day 6 of 28.71 (90% CI: −15.14 to 72.56). Imputation analysis using LOCF also suggested more rapid improvement in SpO_2_/FiO_2_ ratio in the eclitasertib vs. placebo group.

**Table 2:** Change from baseline in SpO_2_/FiO_2_ ratio (efficacy population)

Parameter	Visit	Placebo (*N* = 20)	Eclitasertib 600 mg (*N* = 47)
Change from baseline in SpO_2_/FiO_2_ ratio	Day 02	49.91 (− 24.29 to 124.12)	72.02 (− 1.53 to 145.56)
	Day 03	82.04 (5.68 to 158.39)	97.90 (23.35 to 172.45)
	Day 04	84.91 (5.95 to 163.87)	104.61 (28.83 to 180.39)
	Day 05	102.55 (23.28 to 181.82)	121.79 (45.87 to 197.72)
	Day 06	105.95 (26.73 to 185.18)	134.66 (58.76 to 210.57)
	Day 07	116.97 (36.66 to 197.29)	142.21 (65.78 to 218.63)
	Day 15	150.78 (71.65 to 229.90)	174.65 (98.79 to 250.51)
	**Eclitasertib vs. placebo on Day 07**	**25.24 (− 21.54 to 72.01)**	

N: Number of patients; SpO_2_/FiO_2_: Peripheral oxygen saturation/Fraction of inspired oxygen

Values are point estimates of the mean absolute change from baseline in SpO_2_/FiO_2_ ratio (90% CI). A positive value in the change indicates a larger ratio at the day of measurement than at baseline

During the 28-day study period, eclitasertib treatment was favoured over the placebo in terms of the number of days without need for oxygen support (2.5 days reduction); mean VFD (2.6 days reduction) and mean RFFD (2.6 days reduction) also showed similar trends (Table 3). During the 15-day treatment period, a difference of 1 day in the mean days (SD) without need for oxygen support (placebo: 7.8 [5.3] vs. eclitasertib 600 mg: 8.8 [4.6]), VFDs (12.4 [5.3] vs. 13.9 [4.0]), and RFFDs (12.3 [5.4] vs. 13.9 [4.0]) was observed in the eclitasertib group and placebo group. The proportion of patients receiving anti-thrombotic treatment up to Day 28 was slightly lower in the eclitasertib group (42.1%) than the placebo group (48.8%). Moreover, fewer patients received vasopressor treatment in the eclitasertib group (2.4% vs. 15.8%) than in the placebo group (Table 3).

**Table 3:** Summary of secondary efficacy endpoints (efficacy population)

	Placebo (*N* = 19)	Eclitasertib 600 mg (*N* = 41)	Ratio (95%CI)
**Number of days without need for oxygen Support and alive (days)**			
Mean (SD)	18.0 (10.2)	20.5 (7.7)	0.85 (0.57 to 1.29)
Median	22.0	23.0	
**Number of ventilator-free days and alive (days)**			
Mean (SD)	23.4 (10.0)	26.0 (7.4)	0.70 (0.41 to 1.19)
Median	28.0	28.0	
**Mortality at 28 days, n(%)**	2 (10.5%)	2 (5%)	0.46 (0.07 to 3.06)
**Number of respiratory failure-free days and alive (days)**			
Mean (SD)	23.3 (10.0)	25.9 (7.4)	0.69 (0.41 to 1.16)
Median	28.0	28.0	
**Number (%) of patients receiving treatments up to Day 28**			
Antithrombotics	8 (42.1)	20 (48.8)	
Prophylaxis	8 (42.1)	18 (43.9)	
Adverse event	0	3 (7.3)	
Vasopressor	3 (15.8)	1 (2.4)	

N: Number of patients; SpO_2_/FiO_2_: Peripheral oxygen saturation/Fraction of inspired oxygen

Ratio: Geometric means ratio, except for Mortality where the relative risk ratio is provided

Regarding laboratory markers of severe COVID-19, numerically larger decreases in the baseline-adjusted geometric means of relative changes from baseline were observed in the eclitasertib vs. placebo group for the following: D-dimer on Day 7 only, leukocytes on Day 7 only, neutrophil/lymphocyte ratio on Day 7 eclitasertib at EoT, and LDH on Day 7 and at EoT. Differences in ferritin levels between the eclitasertib and placebo groups were not meaningful (Table 4).

**Table 4:** Summary of laboratory markers of severe COVID-19 (efficacy population)

Parameter	Comparison	Point estimate	90% CI
D-dimer	Eclitasertib vs. placebo on Day 7	0.88	(0.63 to 1.21)
	Eclitasertib vs. placebo at EoT	1.07	(0.73 to 1.58)
Leukocytes	Eclitasertib vs. placebo on Day 7	0.87	(0.73 to 1.03)
	Eclitasertib vs. placebo at EoT	1.03	(0.86 to 1.23)
Lymphocytes	Eclitasertib vs. placebo on Day 7	1.02	(0.75 to 1.38)
	Eclitasertib vs. placebo at EoT	1.03	(0.78 to 1.37)
Neutrophils/Lymphocytes (ratio)	Eclitasertib vs. placebo on Day 7	0.65	(0.42 to 1.00)
	Eclitasertib vs. placebo at EoT	0.67	(0.44 to 1.02)
Ferritin	Eclitasertib vs. placebo on Day 7	0.96	(0.78 to 1.19)
	Eclitasertib vs. placebo at EoT	0.98	(0.77 to 1.24)
Lactate dehydrogenase	Eclitasertib vs. placebo on Day 7	0.80	(0.70 to 0.92)
	Eclitasertib vs. placebo at EoT	0.85	(0.75 to 0.97)

CI: Confidence interval; COVID-19: Coronavirus disease 2019; EoT: End of treatment

Values are expressed as point estimates of the relative change from baseline (geometric means) with two-sided 90% confidence interval. Point estimate: a value lower than 1 indicates a larger decrease from baseline in treatment group than in placebo group. EoT: End of treatment, or discharge/early discontinuation up to Day 15

Relative changes from baseline in peripheral cytokines and biomarkers were analysed for the two treatment groups over time up to EoT (Day 15) (Supp Table 2**)**. The decreases in plasma IL-6, IL-10, and IL-6/IL-10 ratio were numerically greater in the eclitasertib than the placebo group (Supp Fig. 2). The viral load tended to decrease, and the number of negative SARS-CoV-2 tests increased over time in the eclitasertib group compared with the placebo group (Supp Table 3). Eclitasertib was associated with faster improvement on the 7-point ordinal scale vs. placebo (Supp Fig. 3).

In patients with severe COVID-19, after administration of eclitasertib 300 mg BID for up to 14 days, steady state was reached on Day 3 with mean (SD) values of 2025 (783) ng/mL for C_trough_, 5169 (1056) ng/mL for C_max_, and 42,214 (10,949) ng·h/mL for AUC_0–12 h_.

Overall, 4 (5.9%) deaths, all due to COVID-19 complications or worsening of COVID-19, occurred during the study up to Day 28. Two deaths each were reported in the placebo group (10.0%) and the eclitasertib group (4.3%). The most frequently reported treatment-emergent adverse events were gastrointestinal disorders (placebo vs. eclitasertib: 20% vs. 12.8%), condition aggravated/worsened COVID-19 pneumonia (placebo vs. eclitasertib: 20.0% vs. 8.5%), elevated alanine transaminase (placebo vs. eclitasertib: 10.0% vs. 12.8%), and infections (placebo vs. eclitasertib: 25% vs. 8.5%) (Supp Table 4). Most reported treatment-emergent adverse events were considered not related to the study drug by the principal investigators.

## Discussion

To our knowledge, this is the first study to explore the immunomodulatory and clinical effects of a RIPK1 inhibitor (eclitasertib) vs. placebo in patients with severe COVID-19. This study did not demonstrate a statistically significant difference in the selected primary endpoint of relative change in CRP on Day 7 from baseline between the treatment and placebo groups. However, the relative CRP decrease from baseline was numerically greater in the eclitasertib group vs. placebo on Day 7, despite the prespecified primary analysis showing about 50% missing data due to a high proportion of patients recovering and being discharged earlier from the hospital than anticipated. A time-to-event Kaplan-Meier analysis, which is less sensitive to missing data, showed an earlier decrease in CRP for eclitasertib on Day 7; the difference to placebo approached statistical significance (*p* = 0.056). Of note, this effect was observed despite systemic glucocorticoids, known to decrease CRP concentrations, being used in approximately 65% of patients in both groups. Eclitasertib also achieved numerically greater improvement in other inflammatory biomarkers including some of the markers of COVID-19 severity (leukocytes, neutrophil/lymphocyte ratio, LDH, IL-6, IL-8, and IL-6/IL-10) than placebo. Consistent trends toward greater improvements in clinical endpoints were noted in the eclitasertib group than the placebo group with a trend towards faster improvement in oxygenation (SpO_2_/FiO_2_ ratio), along with improvements in SpO_2_ and 7-point clinical scale scores over the treatment period **(**Fig. 3**)**. Moreover, eclitasertib showed slight improvements in VFDs and RFFDs, with a treatment difference of 2.6 days for each compared with placebo.

Current COVID-19 therapies such as baricitinib, dexamethasone, remdesivir, anakinra, and tocilizumab reduce 28-day mortality [10, 24–29]. In the current study, the 28-day mortality was 4.3% (2/47) in the eclitasertib 600 mg group and 10% (2/19) in the placebo group. However, the small sample size precludes any meaningful interpretation of these data and warrants larger, adequately powered studies.

In the current study, the median time to an improvement by at least 2 points on the 7-point clinical scale was also numerically shorter in patients treated with eclitasertib than in those treated with placebo, with a difference of 2 days (*p* = 0.377). The median time to an improvement by one category on the ordinal scale was reported to be 6 days in the baricitinib plus remdesivir group and 8 days in the placebo group (rate ratio: 1.21; 95% CI: 1.06–1.39) [24]. Patients treated with remdesivir had a shorter time to improvement in one or two categories on the ordinal scale from baseline than patients in the placebo group (one-category improvement: median: 7 vs. 9 days and two-category improvement: median: 11 vs. 14 days) [25]. The present study analysed patients not requiring mechanical or high-flow oxygen ventilation at study entry. Thus, the maximum number of VFDs or RFFDs was theoretically 28 days through the study period. A difference of 2 days between eclitasertib and placebo in RFFD is considered clinically relevant [30]. In the current study, the observed mean RFFD was 23.3 days in the placebo group vs. 25.9 days in the eclitasertib group. In our study, new use of mechanical ventilation or extracorporeal membrane oxygenation (ECMO) was observed in 1 (2.4%) patient in the eclitasertib group and 3 (15.8%) patients in the placebo group. The incidence of new use of mechanical ventilation or ECMO in the baricitinib plus remdesivir and placebo group is reported as 10.0% and 15.2%, respectively [24]. The incidence of new use of mechanical ventilation or ECMO in the remdesivir and placebo group is reported to be 13.0% and 23%, respectively [25].

High neutrophil counts and marked lymphopenia (elevated neutrophil/lymphocyte ratio) are associated with severe COVID-19 disease and the risk of developing sepsis with rapid progression. Moreover, a high baseline level of pro-inflammatory biomarkers and an increase in LDH are associated with COVID-19 disease progression and poor outcomes. In the current study, the leukocyte count, neutrophil/lymphocyte ratio, and LDH level as well as plasma IL-6, IL-8 and IL-6/IL-10 showed a trend towards being lower in the eclitasertib than placebo group. The trend towards a decrease in neutrophil/lymphocyte ratio and in levels of pro-inflammatory biomarkers in the eclitasertib group are coherent with the observed lesser use of vasopressors (norepinephrine) in the eclitasertib than the placebo group.

It is to be noted that Xu et al. (2021) recently found evidence of RIPK1 activation in human COVID-19 lung pathological samples, cultured human lung organoids and ACE2 transgenic mice infected by SARS-CoV-2 and as one of the drivers of inflammatory cytokine storm [31]. Strikingly activated RIPK1 were enriched in ciliated epithelial cells in the airways of severe COVID-19 patients which corresponded to elevated levels of proinflammatory cytokines in the BALF, lung and PBMCs in patients as compared to that of control individuals. Inhibition of RIPK1 with Nec-1s reduced mortality and lung viral load while blocking CNS manifestation of SARS-CoV-2 in ACE2 transgenic mice [31]. Inhibition of RIPK1 kinase activity reduced the viral load and inflammation in lung organoids infected with SARS-CoV-2. Mechanistically, in cells with a high viral load, they found that the RNA-dependent RNA polymerase of SARS-CoV-2, NSP12, a highly conserved central component of coronaviral replication and transcription machinery, promoted the activation of RIPK1. In addition, they showed that inhibition of RIPK1 downregulated the transcriptional induction of proinflammatory cytokines and host factors including ACE2 and EGFR that in turn promote viral entry into cells. It was demonstrated that Nec-1s inhibits propagation of NSP12 323P and 323 L variants (that have increased ability to activate RIPK1) SARS-CoV-2 and inflammation [32, 33]. Overall, these data support the rationale for RIPK1 inhibition as a potential therapeutic option for the treatment of COVID-19. While there were no statistically significant differences in primary and key secondary outcomes in this study, numerical differences consistently favoured eclitasertib group over placebo. Whether RIPK1 inhibition can emerge as an effective therapeutic strategy, either alone or in combination with other immunomodulatory therapies, in patients with severe COVID-19 warrants further investigation.

The incidence of AEs was similar between eclitasertib and placebo groups. Notably, the incidence of worsening of COVID-19 and infections and infestations were lower in patients treated with eclitasertib compared with those in the placebo group. The safety data suggest that overall, eclitasertib had a favourable safety profile and was well-tolerated.

The present study with eclitasertib has some limitations inherent with proof-of-mechanism, Phase 1b studies. The number of patients included in the study was limited, leading to low statistical power to detect differences in clinical outcomes. The ability to collect data was limited during the pandemic. The prespecified analysis on Day 7 for the primary endpoint was compromised by about 50% missing data due to the early discharge of patients from the hospital.

## Conclusions

Eclitasertib was well-tolerated. Consistently greater numerical improvements were observed across multiple biomarkers and clinical outcomes for eclitasertib than placebo. Larger confirmatory trials are needed to further assess the clinically significant effects of eclitasertib.

### Electronic supplementary material

Supplementary material

## Data Availability

The qualified researchers may request access to patient-level data and related documents [such as clinical study report, study protocol with any amendments, blank case report form, statistical analysis plan, and dataset specifications]. Patient-level data will be anonymised, and study documents will be redacted to protect the privacy of trial patients. Further details on Sanofi’s data sharing criteria, eligible studies, and process for requesting access can be found at https://vivli.org/.

## Declarations

### This work was previously presented at

European Respiratory Society – 31st Annual Congress (ERS 2021).
