# Supplementary material for: Immunomodulatory and clinical effects of receptor-interacting protein kinase 1 (RIPK1) inhibitor eclitasertib (SAR443122) in patients with severe COVID-19: a phase 1b, randomized, double-blinded, placebo-controlled study
Source: Respir Res. 2024 Feb 28;25:107. doi: 10.1186/s12931-024-02670-z (PMC10903152; doi:10.1186/s12931-024-02670-z)
Supplement: Supplementary file 1 — Supplementary material [file 12931_2024_2670_MOESM1_ESM.docx]

**SUPPLEMENTARY FIGURES AND TABLES**


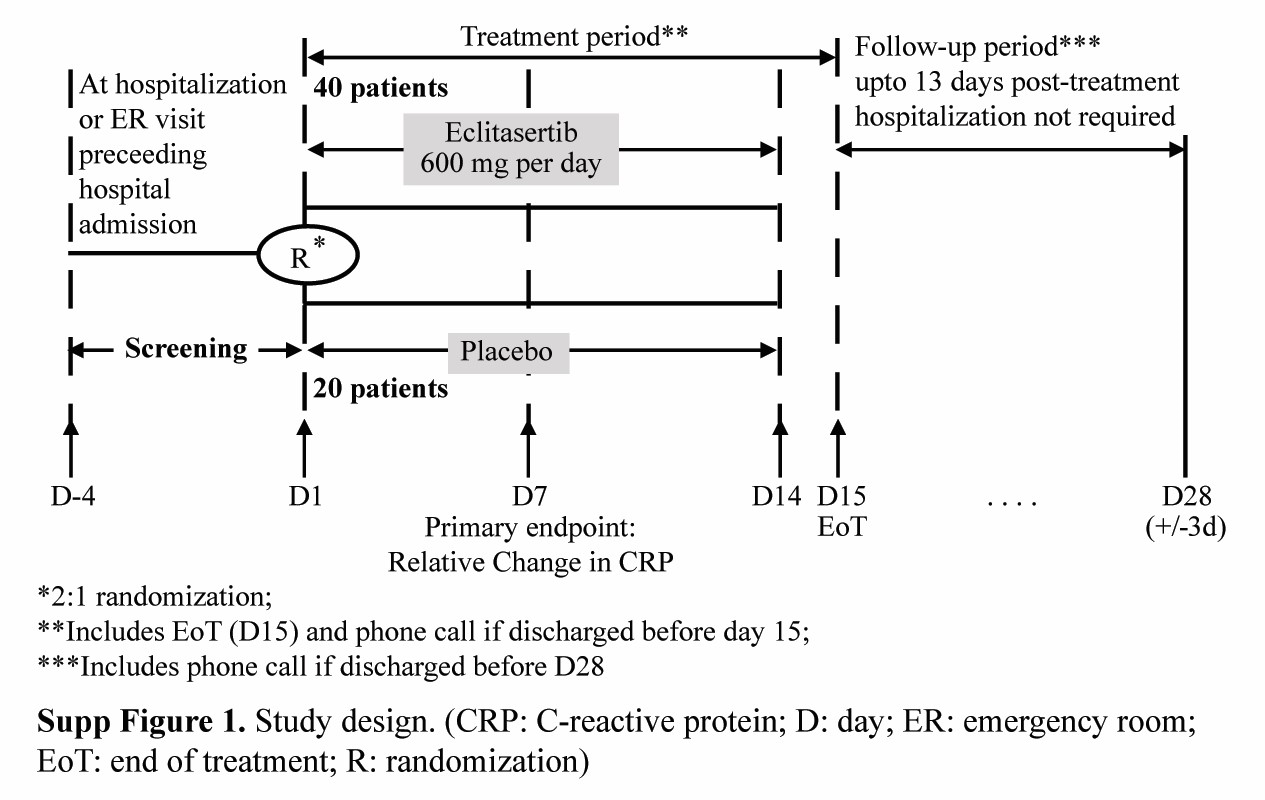


**Supp Figure 1.** Study design. (CRP: C-reactive protein; D: day; ER: emergency room; EoT: end of treatment; R: randomization)


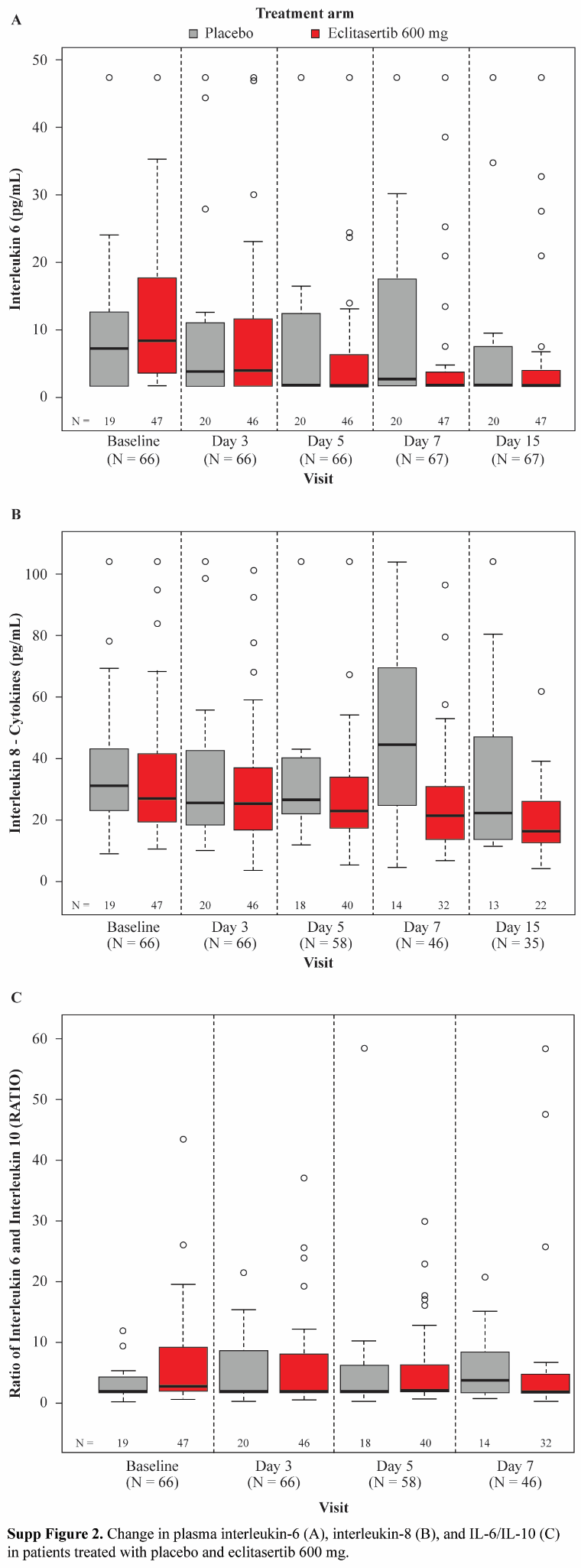


**Supp Figure 2.** Change in plasma interleukin-6 (A), interleukin-8 (B), and IL-6/IL-10 (C) in patients treated with placebo and eclitasertib 600 mg.


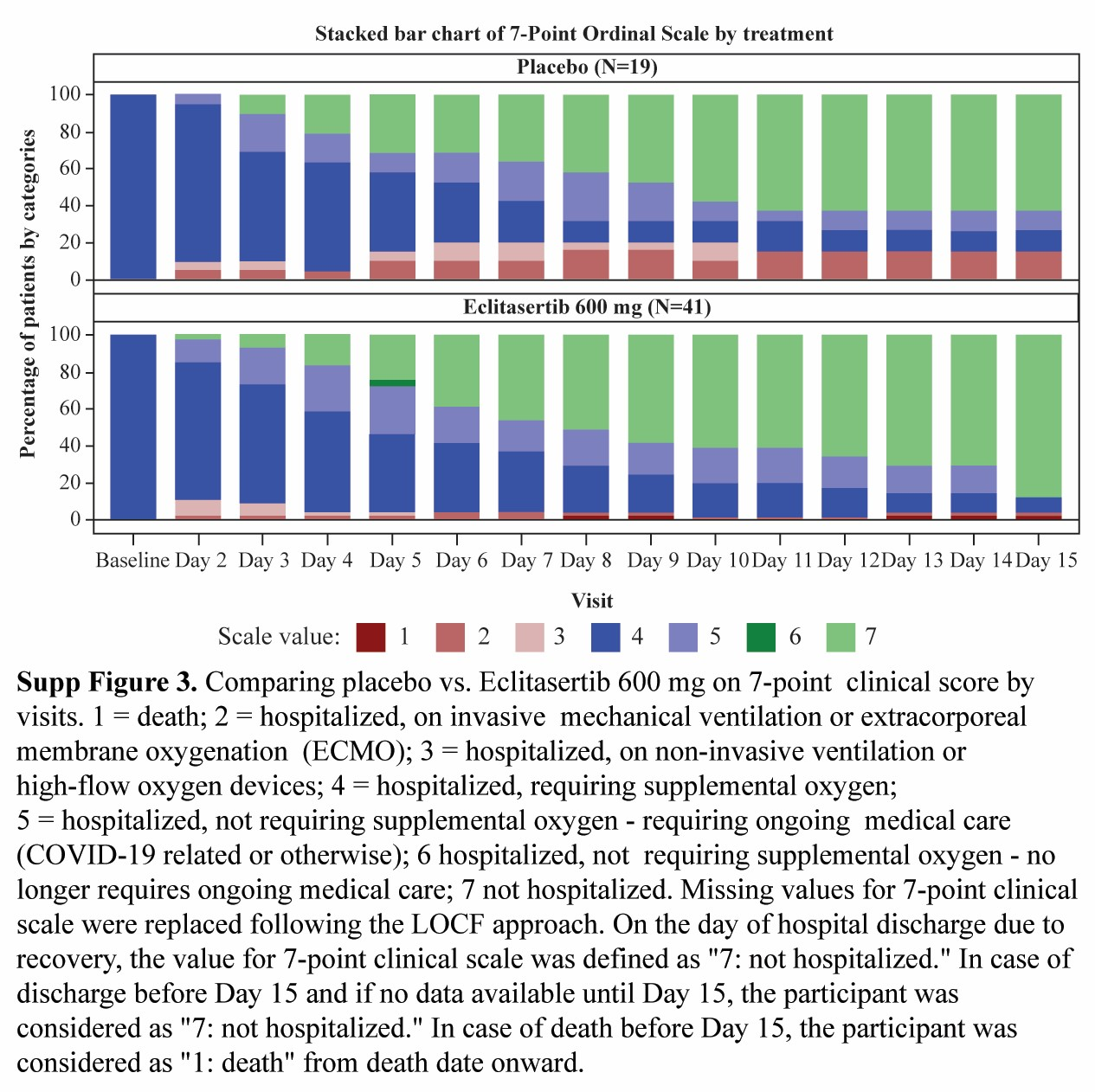


**Supp Figure 3.** Comparing placebo vs. eclitasertib 600 mg on 7-point clinical score by visits. 1 = death; 2 = hospitalised, on invasive mechanical ventilation or extracorporeal membrane oxygenation (ECMO); 3 = hospitalised, on non-invasive ventilation or high-flow oxygen devices; 4 = hospitalised, requiring supplemental oxygen; 5 = hospitalised, not requiring supplemental oxygen - requiring ongoing medical care (COVID-19 related or otherwise); 6 = hospitalised, not requiring supplemental oxygen - no longer requires ongoing medical care; 7 = not hospitalised. Missing values for 7-point clinical scale were replaced following the LOCF approach. On the day of hospital discharge due to recovery, the value for 7-point clinical scale was defined as “7: not hospitalised.” In case of discharge before Day 15 and if no data available until Day 15, the patient was considered as “7: not hospitalised.” In case of death before Day 15, the patient was considered as “1: death” from death date onward.

**Supp Table 1. List of Institutional review board/institutional ethics committee of each study site**

| **Study center no.** | **IRB/IEC name and address** | | **Ethical committee approval number / reference number** | |
| --- | --- | --- | --- | --- |
|  | **Local** | **National** | **Local** | **National** |
| 0320001 | CIE para Ensayos en Farmacologia Clin Prof  Dr Luis M. Zieher  Jose E. Uriburu 774 piso 1, Buenos Aires  C1027AAP  Buenos Aires  Argentina | Not available | DI 2020 4736 | NA |
| 0760001 | EC of Faculdade de Medicina de Sao Jose do  Rio Preto, Av. Brigadeiro Faria Lima, - 5416 - Vila São  Pedro, Sao José Do Rio Preto, 15090-000  Brazil | Not available | 33733220.2.1001.5415 | NA |
| 0760002 | EC of REDE D´OR SÃO LUIZ S.A. Rua Dr. Alceu de Campos Rodrigues, 95 – Vila, Nova Conceição  Sao Paulo, 04544-000  Brazil | Not available | 33733220.2.2002.0087 | NA |
| 0760003 | EC of Hospital de Clinicas de Porto Alegre  Rua Ramiro Barcelos, 2350, 2 andar, sala  2227, Porto Alegre  90035-903, Rio Grande do Sul, Brazil | Not available | 33733220.2.2001.5327 | NA |
| 1520001 | Comité de Ética Científico S.S. M Oriente  Av . Salvador # 364  Providencia Santiago 7500922, Reg Metropolitana de Santiago, Chile | Not available | NA | NA |
| 1520002 | Comite de Etica de la Investigacion S.S.M Sur  Oriente, Av. Concha y Toro # 3459, Puente Alto, Santiago  8207257, Reg Metropolitana de Santiago Chile | Not available | NA | NA |
| 1520003 | SERVICIO DE SALUD METROPOLITANO  SUR COMITE ETICO CIENTIFICO  Santa Rosa 3453, Santiago 8900000, Reg Metropolitana de Santiago Chile | Not available | NA | NA |
|  | Comite de Etica de la Investigacion S.S.M. Sur  Av. Santa Rosa # 3453  Santiago, 8900390  Reg Metropolitana de Santiago - Chile | Not available | NA | NA |
| 4840001 | CB Hospital Universitario "Dr. Jose Eleuterio Gonzalez"  Av. Francisco I. Madero y Gonzalitos S/N, Col.  Mitras Centro.Monterrey  64460 Nuevo León, Mexico | Not available | NMV-00004 | NA |
|  | Comité de Investigación Hospital Universitario "Dr. Jose Eleuterio  Gonzalez" Av. Francisco I. Madero y Av. Gonzalitos S/N  Col. Mitras Centro  Monterrey, 64460  Nuevo León, Mexico | Not available | NMV-00004 | NA |
|  | Comité de Ética Independiente Hospital Universitario "Dr. Jose Eleuterio  Gonzalez" Av. Francisco I. Madero y Av. Gonzalitos S/N  Col. Mitras Centro  Monterrey, 64460  Nuevo León, Mexico | Not available | NMV-00004 | NA |
| 6430001 | Moscow City Independent Ethics Committee 12, build. 2, Minskaya str., Moscow, 121096  Russian Federation | Ethics Board at Ministry of Health of the Russian Federation  3, Rakhmanovsky per., Moscow, 127994  Russian Federation | №58 dd. 08 Jul 2020 | №233 dd. 25 Jun 2020 |
|  | Ethics Board at Ministry of Health of the Russian Federation  3, Rakhmanovsky per., Moscow, 127994  Russian Federation | Ethics Board at Ministry of Health of the Russian Federation  3, Rakhmanovsky per., Moscow, 127994  Russian Federation | NA | №233 dd. 25 Jun 2020 |
| 6430002 | Local Ethics Committee of the City Clinical  Hospital #52  3, Pekhotnaya str., Moscow, 123182  Russian Federation | Ethics Board at Ministry of Health of the Russian Federation  3, Rakhmanovsky per., Moscow, 127994  Russian Federation | №07вн-02/0620 dd. 06 Jul 2020 | №233 dd. 25 Jun 2020 |
|  | Ethics Board at Ministry of Health of the  Russian Federation  3, Rakhmanovsky per.,  Moscow, 127994  Russian Federation | Ethics Board at Ministry of Health of the Russian Federation  3, Rakhmanovsky per., Moscow, 127994 Russian Federation | NA | №233 dd. 25 Jun 2020 |
| IRB/IEC: Institutional review board/institutional ethics committee; NA: Not applicable | | | | |

**Supp Table 2. Summary of pro-inflammatory cytokines and RIPK1-correlated cytokines/chemokines on Day 7 (safety population)**

| **Biomarker** | **Placebo  Fold-Change (n)** | **Placebo  P-value / FDR** | **Eclitasertib 600 mg  Fold-Change (n)** | **Eclitasertib 600 mg  P-value / FDR** | **Eclitasertib 600 mg vs. Placebo Fold-Change (n)** | **Eclitasertib 600 mg vs. Placebo P-value / FDR** |  |
| --- | --- | --- | --- | --- | --- | --- | --- |
| Tumor necrosis factor alpha (pg/mL) | 0.87 (19) | 0.154 / 0.2 | 0.85 (47) | 0.0113 / 0.0146 | 0.98 (66) | 0.86 / 0.942 |  |
| Chemokine (C-C motif) ligand 13 (pg/mL) | 1.15 (19) | 0.0612 / 0.133 | 1.31 (41) | 5.315e-07 / 1.152e-06 | 1.15 (60) | 0.115 / 0.452 |  |
| Chemokine (C-C motif) ligand 17 (pg/mL) | 1.55 (19) | 3.791e-05 / 0.000164 | 1.56 (41) | 1.334e-08 / 4.337e-08 | 1 (60) | 0.979 / 0.979 |  |
| Monocyte chemotactic protein 1 (pg/mL) | 0.82 (19) | 0.131 / 0.19 | 0.69 (41) | 0.000137 / 0.000254 | 0.85 (60) | 0.304 / 0.495 |  |
| Macrophage-derived chemokine (pg/mL) | 0.88 (19) | 0.347 / 0.408 | 1.05 (41) | 0.572 / 0.572 | 1.2 (60) | 0.275 / 0.495 |  |
| Interferon gamma (pg/mL) | 0.43 (19) | 3.942e-07 / 2.562e-06 | 0.44 (47) | 3.096e-12 / 1.342e-11 | 1.03 (66) | 0.87 / 0.942 |  |
| Ratio of interleukin 6 and interleukin 10 (ratio) | 1.01 (19) | 0.971 / 0.971 | 0.87 (47) | 0.415 / 0.449 | 0.86 (66) | 0.643 / 0.929 |  |
| Macrophage inflammatory protein 1 beta (pg/mL) | 1.26 (19) | 0.0108 / 0.0281 | 1.07 (41) | 0.233 / 0.276 | 0.85 (60) | 0.139 / 0.452 |  |
| Interleukin 10 (pg/mL) | 0.58 (19) | 0.000159 / 0.000515 | 0.48 (47) | 2.311e-12 / 1.342e-11 | 0.82 (66) | 0.213 / 0.462 |  |
| Interleukin 6 (pg/mL) | 0.64 (19) | 0.0886 / 0.144 | 0.4 (47) | 4.891e-07 / 1.152e-06 | 0.63 (66) | 0.129 / 0.452 |  |
| Interleukin 8 – cytokines (pg/mL) | 0.88 (19) | 0.377 / 0.408 | 0.71 (47) | 0.000216 / 0.000351 | 0.8 (66) | 0.181 / 0.462 |  |
| Eotaxin-1 (pg/mL) | 1.17 (20) | 0.0888 / 0.144 | 1.21 (45) | 0.00264 / 0.00381 | 1.03 (65) | 0.766 / 0.942 |  |
| Chemokine (C-X-C motif) ligand 10 (pg/mL) | 0.37 (19) | 2.206e-08 / 2.868e-07 | 0.26 (37) | 2.765e-17 / 3.594e-16 | 0.7 (56) | 0.066 / 0.452 |  |
| FDR: False discovery rate; IQR: Inter-quartile range; LLOQ: Lower limit of quantitation; Q3: Third quartile  Note: n = Number of patients with baseline and Day 7 assessments. Baseline is defined as the D1 predose assessment value. Values below LLOQ are replaced by LLOQ/2. Outlier values higher than Q3 + 3 IQR are imputed by Q3 + 3 IQR. Missing data are imputed by last observation carried forward (LOCF) method if at least one baseline and one post-baseline values were available. Unscheduled and discharge before Day 15 (treatment period) visits are re-allocated to study visits according to their study day. Linear fixed effect model with treatment as fixed effect, and baseline as covariate on log-transformed absolute change from baseline. Fold-Changes are calculated using exponential of log-least squared means in each treatment arm, and exponential of log-least squared means difference between arms. FDR = False discovery rate adjusted *p*-value using the Benjamini-Hochberg procedure. | | | | | | | |
|  | | | | | | | |
|  | | | | | | | |

**Supp Table 3. Summary of SARS-CoV-2 viral load in blood (efficacy population)**

|  | **Placebo (N = 19)** | **Eclitasertib 600 mg (N = 41)** | **All (N = 60)** |  |
| --- | --- | --- | --- | --- |
| **Day 01** |  |  |  |  |
| N | 16 | 33 | 49 |  |
| Inconclusive | 1 (5.3) | 1 (2.4) | 2 (3.3) |  |
| No SARS-CoV-2 detected | 4 (21.1) | 11 (26.8) | 15 (25.0) |  |
| <1660 cp/mL SARS-CoV-2 | 4 (21.1) | 14 (34.1) | 18 (30.0) |  |
| Positive result | 7 (36.8) | 7 (17.1) | 14 (23.3) |  |
|  |  |  |  |  |
| Positive result (copies/mL) |  |  |  |  |
| N | 7 | 7 | 14 |  |
| Mean (SD) | 14677.0 (25730.0) | 30217.6 (42992.8) | 22447.3 (34981.0) |  |
| Median | 4751.0 | 7560.0 | 6155.5 |  |
| Q1; Q3 | 2043.0; 10367.0 | 1960.0; 78562.0 | 2043.0; 14455.0 |  |
| Min; Max | 2018; 72532 | 1759; 105034 | 1759; 105034 |  |
|  |  |  |  |  |
| **Day 03** |  |  |  |  |
| n | 15 | 34 | 49 |  |
| Inconclusive | 2 (10.5) | 2 (4.9) | 4 (6.7) |  |
| No SARS-CoV-2 detected | 4 (21.1) | 16 (39.0) | 20 (33.3) |  |
| <1660 cp/mL SARS-CoV-2 | 4 (21.1) | 12 (29.3) | 16 (26.7) |  |
| Positive result | 5 (26.3) | 4 (9.8) | 9 (15.0) |  |
|  |  |  |  |  |
| Positive result (copies/mL) |  |  |  |  |
| n | 5 | 4 | 9 |  |
| Mean (SD) | 7560.4 (7482.7) | 7505.0 (6425.8) | 7535.8 (6593.9) |  |
| Median | 3603.0 | 6332.5 | 3603.0 |  |
| Q1; Q3 | 2434.0; 10316.0 | 2350.5; 12659.5 | 2434.0; 10316.0 |  |
| Min; Max | 1938; 19511 | 1784; 15571 | 1784; 19511 |  |
|  |  |  |  |  |
| **DAY 07** |  |  |  |  |
| n | 10 | 16 | 26 |  |
| Inconclusive | 0 | 2 (4.9) | 2 (3.3) |  |
| No SARS-CoV-2 detected | 6 (31.6) | 12 (29.3) | 18 (30.0) |  |
| <1660 cp/mL SARS-CoV-2 | 1 (5.3) | 1 (2.4) | 2 (3.3) |  |
| Positive result | 3 (15.8) | 1 (2.4) | 4 (6.7) |  |
|  |  |  |  |  |
| Positive result (copies/mL) |  |  |  |  |
| n | 3 | 1 | 4 |  |
| Mean (SD) | 12937.3 (18742.0) | 6240.0 (NC) | 11263.0 (15664.9) |  |
| Median | 2549.0 | 6240.0 | 4394.5 |  |
| Q1 ; Q3 | 1690.0; 34573.0 | 6240.0; 6240.0 | 2119.5; 20406.5 |  |
| Min ; Max | 1690; 34573 | 6240; 6240 | 1690; 34573 |  |
|  |  |  |  |  |
| **EoT** |  |  |  |  |
| n | 17 | 33 | 50 |  |
| Inconclusive | 0 | 1 (2.4) | 1 (1.7) |  |
| No SARS-CoV-2 detected | 13 (68.4) | 28 (68.3) | 41 (68.3) |  |
| <1660 cp/mL SARS-CoV-2 | 3 (15.8) | 4 (9.8) | 7 (11.7) |  |
| Positive result | 1 (5.3) | 0 | 1 (1.7) |  |
|  |  |  |  |  |
| Positive result (copies/mL) |  |  |  |  |
| n | 1 | 0 | 1 |  |
| Mean (SD) | 3609.0 (NC) | NC (NC) | 3609.0 (NC) |  |
| Median | 3609.0 | NC | 3609.0 |  |
| Q1; Q3 | 3609.0; 3609.0 | NC; NC | 3609.0; 3609.0 |  |
| Min; Max | 3609; 3609 | NC; NC | 3609; 3609 |  |
| Min: Minimum; Max: Maximum; n: number of patients with non-missing data; NC: Not calculated; Q1: First quartile; Q3: Third quartile; SARS-COV-2: severe acute respiratory syndrome coronavirus 2; SD: Standard deviation  Note: Baseline is defined as the D1 predose assessment value; cp/mL: copies/mL. Some samples were not analysed by the laboratory due to “insufficient quantity” or “questionable integrity”. | | | | |

**Supp Table 4. Number (%) of patients with TEAE(s) by primary SOC and PT (safety population)**

| **Primary System Organ Class Preferred Term, n (%)** | **Placebo (N = 20)** | **Eclitasertib 600 mg (N = 47)** | **Relative risk ratio** | **95%CI** |  |  |  |  |
| --- | --- | --- | --- | --- | --- | --- | --- | --- |
| Any class | 10 (50.0) | 24 (51.1) | 1.02 | (0.61 to 1.72) |  |  |  |  |
|  |  |  | . |  |  |  |  |  |
| **Infections and infestations** | 5 (25.0) | 4 (8.5) | 0.34 | (0.10 to 1.14) |  |  |  |  |
| Bacterial infection | 1 (5.0) | 1 (2.1) | 0.43 | (0.03 to 6.47) |  |  |  |  |
| Pneumonia bacterial | 0 | 1 (2.1) | . | (. , .) |  |  |  |  |
| Pseudomembranous colitis | 0 | 1 (2.1) | . | (. , .) |  |  |  |  |
| Pseudomonas infection | 0 | 1 (2.1) | . | (. , .) |  |  |  |  |
| Furuncle | 1 (5.0) | 0 | 0 | (. , .) |  |  |  |  |
| Pneumonia | 1 (5.0) | 0 | 0 | (. , .) |  |  |  |  |
| Tracheitis | 1 (5.0) | 0 | 0 | (. , .) |  |  |  |  |
| Tracheobronchitis | 1 (5.0) | 0 | 0 | (. , .) |  |  |  |  |
|  |  |  | . |  |  |  |  |  |
| **Blood and lymphatic system disorders** | 2 (10.0) | 1 (2.1) | 0.21 | (0.02 to 2.22) |  |  |  |  |
| Anaemia | 2 (10.0) | 1 (2.1) | 0.21 | (0.02 to 2.22) |  |  |  |  |
|  |  |  | . |  |  |  |  |  |
| **Immune system disorders** | 0 | 1 (2.1) | . | (. , .) |  |  |  |  |
| Drug hypersensitivity | 0 | 1 (2.1) | . | (. , .) |  |  |  |  |
|  |  |  | . |  |  |  |  |  |
| **Metabolism and nutrition disorders** | 1 (5.0) | 3 (6.4) | 1.28 | (0.14 to 11.54) |  |  |  |  |
| Dehydration | 0 | 1 (2.1) | . | (. , .) |  |  |  |  |
| Hyperglycaemia | 0 | 1 (2.1) | . | (. , .) |  |  |  |  |
| Hypoglycaemia | 0 | 1 (2.1) | . | (. , .) |  |  |  |  |
| Hypophosphatemia | 0 | 1 (2.1) | . | (. , .) |  |  |  |  |
| Metabolic acidosis | 1 (5.0) | 0 | 0 | (. , .) |  |  |  |  |
|  |  |  | . |  |  |  |  |  |
| **Psychiatric disorders** | 0 | 1 (2.1) | . | (. , .) |  |  |  |  |
| Anxiety disorder | 0 | 1 (2.1) | . | (. , .) |  |  |  |  |
|  |  |  | . |  |  |  |  |  |
| **Nervous system disorders** | 2 (10.0) | 0 | 0 | (. , .) |  |  |  |  |
| Cerebral ischaemia | 1 (5.0) | 0 | 0 | (. , .) |  |  |  |  |
| Encephalopathy | 1 (5.0) | 0 | 0 | (. , .) |  |  |  |  |
| Psychomotor hyperactivity | 1 (5.0) | 0 | 0 | (. , .) |  |  |  |  |
|  |  |  | . |  |  |  |  |  |
| **Cardiac disorders** | 2 (10.0) | 0 | 0 | (. , .) |  |  |  |  |
| Cardiac arrest | 1 (5.0) | 0 | 0 | (. , .) |  |  |  |  |
| Tachycardia paroxysmal | 1 (5.0) | 0 | 0 | (. , .) |  |  |  |  |
|  |  |  | . |  |  |  |  |  |
| **Vascular disorders** | 0 | 3 (6.4) | . | (. , .) |  |  |  |  |
| Hypertension | 0 | 1 (2.1) | . | (. , .) |  |  |  |  |
| Peripheral artery thrombosis | 0 | 1 (2.1) | . | (. , .) |  |  |  |  |
| Venous thrombosis limb | 0 | 1 (2.1) | . | (. , .) |  |  |  |  |
|  |  |  | . |  |  |  |  |  |
| **Respiratory, thoracic, and mediastinal disorders** | 3 (15.0) | 4 (8.5) | 0.57 | (0.14 to 2.31) |  |  |  |  |
| Dyspnoea | 0 | 1 (2.1) | . | (. , .) |  |  |  |  |
| Emphysema | 0 | 1 (2.1) | . | (. , .) |  |  |  |  |
| Oropharyngeal pain | 0 | 1 (2.1) | . | (. , .) |  |  |  |  |
| Pulmonary embolism | 0 | 1 (2.1) | . | (. , .) |  |  |  |  |
| Respiratory disorder | 0 | 1 (2.1) | . | (. , .) |  |  |  |  |
| Noninfective bronchitis | 1 (5.0) | 0 | 0 | (. , .) |  |  |  |  |
| Pleural effusion | 1 (5.0) | 0 | 0 | (. , .) |  |  |  |  |
| Pneumomediastinum | 2 (10.0) | 0 | 0 | (. , .) |  |  |  |  |
| Pneumothorax | 1 (5.0) | 0 | 0 | (. , .) |  |  |  |  |
| Respiratory failure | 1 (5.0) | 0 | 0 | (. , .) |  |  |  |  |
|  |  |  | . |  |  |  |  |  |
| **Gastrointestinal disorders** | 4 (20.0) | 6 (12.8) | 0.64 | (0.20 to 2.02) |  |  |  |  |
| Diarrhoea | 1 (5.0) | 4 (8.5) | 1.7 | (0.20 to 14.29) |  |  |  |  |
| Constipation | 0 | 1 (2.1) | . | (. , .) |  |  |  |  |
| Flatulence | 0 | 1 (2.1) | . | (. , .) |  |  |  |  |
| Nausea | 1 (5.0) | 1 (2.1) | 0.43 | (0.03 to 6.47) |  |  |  |  |
| Dyspepsia | 1 (5.0) | 0 | 0 | (. , .) |  |  |  |  |
| Gastritis | 1 (5.0) | 0 | 0 | (. , .) |  |  |  |  |
| Gastroesophageal sphincter insufficiency | 1 (5.0) | 0 | 0 | (. , .) |  |  |  |  |
| Oesophageal ulcer | 1 (5.0) | 0 | 0 | (. , .) |  |  |  |  |
| Oesophagitis | 1 (5.0) | 0 | 0 | (. , .) |  |  |  |  |
| Pneumoperitoneum | 1 (5.0) | 0 | 0 | (. , .) |  |  |  |  |
| Vomiting | 1 (5.0) | 0 | 0 | (. , .) |  |  |  |  |
|  |  |  | . |  |  |  |  |  |
| **Hepatobiliary disorders** | 1 (5.0) | 0 | 0 | (. , .) |  |  |  |  |
| Cholelithiasis | 1 (5.0) | 0 | 0 | (. , .) |  |  |  |  |
|  |  |  | . |  |  |  |  |  |
| **Skin and subcutaneous tissue disorders** | 2 (10.0) | 0 | 0 | (. , .) |  |  |  |  |
| Subcutaneous emphysema | 2 (10.0) | 0 | 0 | (. , .) |  |  |  |  |
|  |  |  | . |  |  |  |  |  |
| **Musculoskeletal and connective tissue disorders** | 0 | 1 (2.1) | . | (. , .) |  |  |  |  |
| Back pain | 0 | 1 (2.1) | . | (. , .) |  |  |  |  |
|  |  |  | . |  |  |  |  |  |
| **Renal and urinary disorders** | 2 (10.0) | 0 | 0 | (. , .) |  |  |  |  |
| Renal cyst | 1 (5.0) | 0 | 0 | (. , .) |  |  |  |  |
| Renal impairment | 1 (5.0) | 0 | 0 | (. , .) |  |  |  |  |
|  |  |  | . |  |  |  |  |  |
| **Reproductive system and breast disorders** | 1 (5.0) | 0 | 0 | (. , .) |  |  |  |  |
| Ovarian cyst | 1 (5.0) | 0 | 0 | (. , .) |  |  |  |  |
|  |  |  | . |  |  |  |  |  |
| **General disorders and administration site conditions** | 4 (20.0) | 6 (12.8) | 0.64 | (0.20 to 2.02) |  |  |  |  |
| Condition aggravated | 4 (20.0) | 4 (8.5) | 0.43 | (0.12 to 1.54) |  |  |  |  |
| Chest discomfort | 0 | 1 (2.1) | . | (. , .) |  |  |  |  |
| Fatigue | 0 | 1 (2.1) | . | (. , .) |  |  |  |  |
| Non-cardiac chest pain | 0 | 1 (2.1) | . | (. , .) |  |  |  |  |
| Pyrexia | 0 | 1 (2.1) | . | (. , .) |  |  |  |  |
| Vessel puncture site phlebitis | 0 | 1 (2.1) | . | (. , .) |  |  |  |  |
|  |  |  | . |  |  |  |  |  |
| **Investigations** | 4 (20.0) | 6 (12.8) | 0.64 | (0.20 to 2.02) |  |  |  |  |
| Alanine aminotransferase increased | 2 (10.0) | 6 (12.8) | 1.28 | (0.28 to 5.79) |  |  |  |  |
| Aspartate aminotransferase increased | 0 | 1 (2.1) | . | (. , .) |  |  |  |  |
| Blood pressure increased | 1 (5.0) | 0 | 0 | (. , .) |  |  |  |  |
| Transaminases increased | 1 (5.0) | 0 | 0 | (. , .) |  |  |  |  |
|  |  |  | . |  |  |  |  |  |
| **Injury, poisoning, and procedural complications** | 1 (5.0) | 1 (2.1) | 0.43 | (0.03 to 6.47) |  |  |  |  |
| Arterial injury | 0 | 1 (2.1) | . | (. , .) |  |  |  |  |
| Procedural pneumothorax | 1 (5.0) | 0 | 0 | (. , .) |  |  |  |  |
| n (%): number and percentage of patients with at least one TEAE; PT: Preferred term; SOC: System Organ Class; TEAE: Treatment-emergent adverse event  Preferred term: Condition Aggravated in General disorders and administration site conditions corresponds to worsening of COVID-19. | | | | | | |  |  |
